# The Domains of Human Nutrition: The Importance of Nutrition Education in Academia and Medical Schools

**DOI:** 10.3389/fnut.2017.00002

**Published:** 2017-02-22

**Authors:** Lorenzo M. Donini, Francesco Leonardi, Mariangela Rondanelli, Giuseppe Banderali, Maurizio Battino, Enrico Bertoli, Alessandra Bordoni, Furio Brighenti, Riccardo Caccialanza, Giulia Cairella, Antonio Caretto, Hellas Cena, Manuela Gambarara, Maria Gabriella Gentile, Marcello Giovannini, Lucio Lucchin, Pietro Migliaccio, Francesco Nicastro, Fabrizio Pasanisi, Luca Piretta, Danilo Radrizzani, Carla Roggi, Giuseppe Rotilio, Luca Scalfi, Roberto Vettor, Federico Vignati, Nino C. Battistini, Maurizio Muscaritoli

**Affiliations:** ^1^Università di Roma La Sapienza – on behalf of the Italian Society of Parenteral and Enteral Nutrition (SINPE), Rome, Italy; ^2^Azienda Ospedale “Cannizzaro”, Catania – on behalf of the Italian Federation of Nutrition Societies (FeSIN), Catania, Italy; ^3^Università di Pavia – on behalf of the Italian Society of Parenteral and Enteral Nutrition (SINPE), Pavia, Italy; ^4^ASST Santi Paolo e Carlo Presidio Ospedaliero San Carlo, Milano – on behalf of the Italian Society of Human Pediatric Nutrition (SINUPE), Milano, Italy; ^5^Università Politecnica delle Marche – on behalf of the Italian Dietetic Association (ADI), Ancona, Italy; ^6^Università di Ancona – on behalf of the Italian Dietetic Association (ADI), Ancona, Italy; ^7^Università di Bologna – on behalf of the Italian Society of Human Nutrition (SINU), Bologna, Italy; ^8^Università di Parma – on behalf of the Italian Society of Human Nutrition (SINU), Parma, Italy; ^9^Fondazione IRCCS Policlinico San Matteo, Pavia – on behalf of the Italian Society of Parenteral and Enteral Nutrition (SINPE), Pavia, Italy; ^10^ASL RMB, Rome – on behalf of the Italian Society of Human Nutrition (SINU), Rome, Italy; ^11^Perrino Hospital, Brindisi – on behalf of the Italian Dietetic Association (ADI), Brindisi, Italy; ^12^Università di Pavia, Italy; ^13^Bambino Gesu Children Hospital, Roma – on behalf of the Italian Society of Human Pediatric Nutrition (SINUPE), Rome, Italy; ^14^Niguarda Hospital, Milan – on behalf of the Italian Dietetic Association (ADI), Milan, Italy; ^15^University of Milan – on behalf of the Italian Society of Human Pediatric Nutrition (SINUPE), Milan, Italy; ^16^Regional General Hospital, Bolzano – on behalf of the Italian Dietetic Association (ADI), Bolzano, Italy; ^17^On behalf of the Italian Society of Food Science (SISA), Rome, Italy; ^18^Università di Bari – on behalf of the Italian Society of Food Science (SISA), Bari, Italy; ^19^Università degli Studi di di Napoli Federico II – on behalf of the Italian Society of Human Nutrition (SINU), Napoli, Italy; ^20^Università di Roma La Sapienza – on behalf of the Italian Society of Food Science (SISA), Rome, Italy; ^21^AO Ospedale Civile di Legnano – on behalf of the Italian Society of Parenteral and Enteral Nutrition (SINPE), Legnano, Italy; ^22^Università di Pavia, Italy; ^23^Università di Roma Tor Vergata – on behalf of the Federation of Italian Nutrition Societies (FeSIN), Rome, Italy; ^24^Università degli Studi di Napoli Federico II – on behalf of the Italian Society of Human Nutrition (SINU), Napoli, Italy; ^25^Università di Padova – on behalf of the Italian Society of Obesity (SIO), Padova, Italy; ^26^Niguarda Hospital, Milano – on behalf of the Italian Society of Obesity (SIO), Milan, Italy; ^27^University of Modena and Reggio Emilia – on behalf of the Italian Society of Human Nutrition (SINU), Modena, Italy

**Keywords:** human nutrition, academic training, basic nutrition, applied nutrition, clinical nutrition

## Abstract

Human nutrition encompasses an extremely broad range of medical, social, commercial, and ethical domains and thus represents a wide, interdisciplinary scientific and cultural discipline. The high prevalence of both disease-related malnutrition and overweight/obesity represents an important risk factor for disease burden and mortality worldwide. It is the opinion of Federation of the Italian Nutrition Societies (FeSIN) that these two sides of the same coin, with their sociocultural background, are related to a low “nutritional culture” secondary, at least in part, to an insufficient academic training for health-care professionals (HCPs). Therefore, FeSIN created a study group, composed of delegates of all the federated societies and representing the different HCPs involved in human nutrition, with the aim of identifying and defining the domains of human nutrition in the attempt to more clearly define the cultural identity of human nutrition in an academically and professionally oriented perspective and to report the conclusions in a position paper. Three main domains of human nutrition, namely, basic nutrition, applied nutrition, and clinical nutrition, were identified. FeSIN has examined the areas of knowledge pertinent to human nutrition. Thirty-two items were identified, attributed to one or more of the three domains and ranked considering their diverse importance for academic training in the different domains of human nutrition. Finally, the study group proposed the attribution of the different areas of knowledge to the degree courses where training in human nutrition is deemed necessary (e.g., schools of medicine, biology, nursing, etc.). It is conceivable that, in the near future, a better integration of the professionals involved in the field of human nutrition will eventually occur based on the progressive consolidation of knowledge, competence, and skills in the different areas and domains of this discipline.

## Introduction

Since 2004, the WHO projections alerted institutions and the international scientific community about the worrisome patterns of rising mortality rates due to non-transmissible pathologies such as tumors and cardiovascular disease. The “Global health risks: mortality and burden of disease attributable to selected major risks” document (1) lists the risk factors related to dietary-behavioral components, namely, obesity, hypertension, hypercholesterolemia, hyperglycemia, alcohol consumption, low fruit and vegetable intake, sedentary lifestyle, disease-related anorexia and anorexia nervosa, and alteration of metabolism during diseases. More than 60% of the overall disease burden (defined as the number of years lost due to ill-health, disability, or early death: disability-adjusted life years), about 60% of cardiovascular deaths, and 35% of tumor deaths may be attributed to dietary-behavioral components (2–6).

On the other hand, the high rates of undernutrition and in particular of *disease-related malnutrition*, averaging 35% (7), have remained unchanged since the 1970s, despite repeated and unequivocal evidence in the international literature and Council of Europe resolution and recommendations (8). This “disease within a disease,” with its iatrogenic component, causes high rates of complications, mortality, and costs—about 12% or more of hospital expenditure—and it is too often underestimated or not recognized and not diagnosed at all (9).

In spite of their high prevalence, there is a great neglect regarding the diagnosis and treatment of malnutrition (over- and undernutrition). This is most likely related to a number of factors, among which the paucity of physicians and health-care professionals (HCPs) who are full-time dedicated to clinical nutrition, to the low priority given to nutritional activities by other disciplines in the competition for budget in the hospitals, difficulties in reimbursement of nutritional support by the health-care systems of insurance companies (10).

Moreover, sociocultural aspects seem to hinder nutritional aspects related to health status, in particular in developed countries. Obesity is still frequently not recognized as a disease state, while the issue of undernutrition in elderly or obese subjects is largely overlooked. The biological significance of food in the collective imagination has been lost in favor of its hedonistic aspects.

Lay people are led to consider nutrients regardless of the food matrix and to attribute them biological functions in spite of any other nutritional, clinical, functional, or environmental consideration.

It is the opinion of the Federation of the Italian Nutrition Societies (FeSIN) that this confusion in the nutritional scenario could be overcome by improving the academic training in human nutrition.

**Figure 1:**
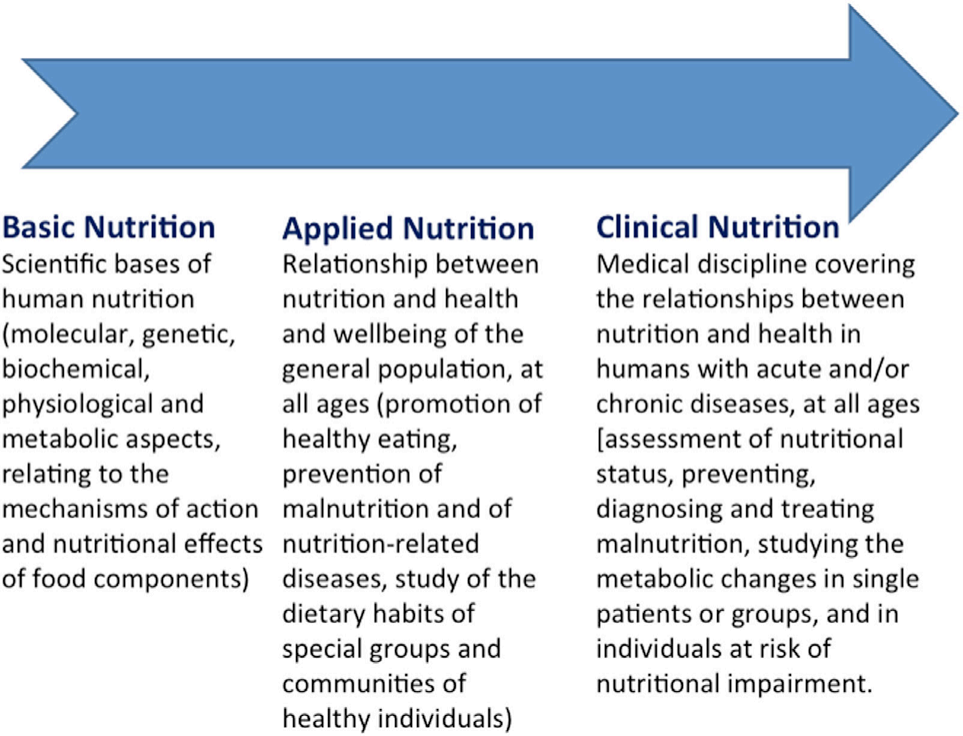
**The continuum of knowledge in human nutrition**.

Human nutrition encompasses an extremely broad range of medical, social, commercial, and ethical domains and thus represents a wide, interdisciplinary scientific and cultural discipline (11, 12) (Figure 1). Human nutrition is an intrinsically complex topic, ranging from agriculture and zootechnics, to food technology, from nutrition in different physiological states (growth, pregnancy, breast-feeding, aging), to the nutritional approach to acute and chronic diseases, from birth to the end of life (13, 14). Therefore, the gray line separating the purely physiological and cultural aspects from the specifically medical domains of human nutrition is extremely thin, and this partly explains why the training offered today is still qualitatively and quantitatively inappropriate to target the different professionals involved in the field of human nutrition (15, 16).

Conceptually, training for human nutrition should feature in different degree courses, with training programs calibrated to specific professional requirements (17). With some exceptions, however, this training is inconsistent in the various academic courses worldwide and the appropriate teaching of knowledge–competencies–skills is unevenly delivered (18). In some cases, there may be no training at all in human nutrition, even where it would be logical to expect it. This is true, for instance, in many study courses in biology and pharmacy. Last, but not least, teaching of human nutrition is generally insufficient even within medical education (19, 20), particularly as regard the clinical aspects, which is surprising, considering the importance of nutrition in relation to both prevention and therapy of diseases (21, 22).

In drafting this position paper, the FeSIN study group’s main goal was to identify the criteria and guiding concepts to be applied in academic training in human nutrition. This paper offers some short comments on the organization of university training in Italy, and briefly, it identifies the subject matter that should characterize the core of human nutrition training for HCPs. To conclude, this position document proposes recommendations that summarize the main points of the document.

## Materials and Methods

For the purposes of this paper, a working group was created by FeSIN composed of delegates of all the federated societies and representing the different HCPs involved in human nutrition. Through online and face-to-face brainstorming meetings, held from November 2013 to December 2015, the domains of human nutrition were identified and defined, together with the “areas of knowledge” pertinent to human nutrition. The definition and characteristics of these items were progressively refined based on the recursive comments of the group participants. The process for the production of the present position paper begun in January 2016. The manuscript draft was then circulated among the group participants. An agreement on the final version of the document and the endorsement by FeSIN executive committee was reached in June 2016.

## Results

### Identification and Definition of the Domains of Human Nutrition

**Figure 2:**
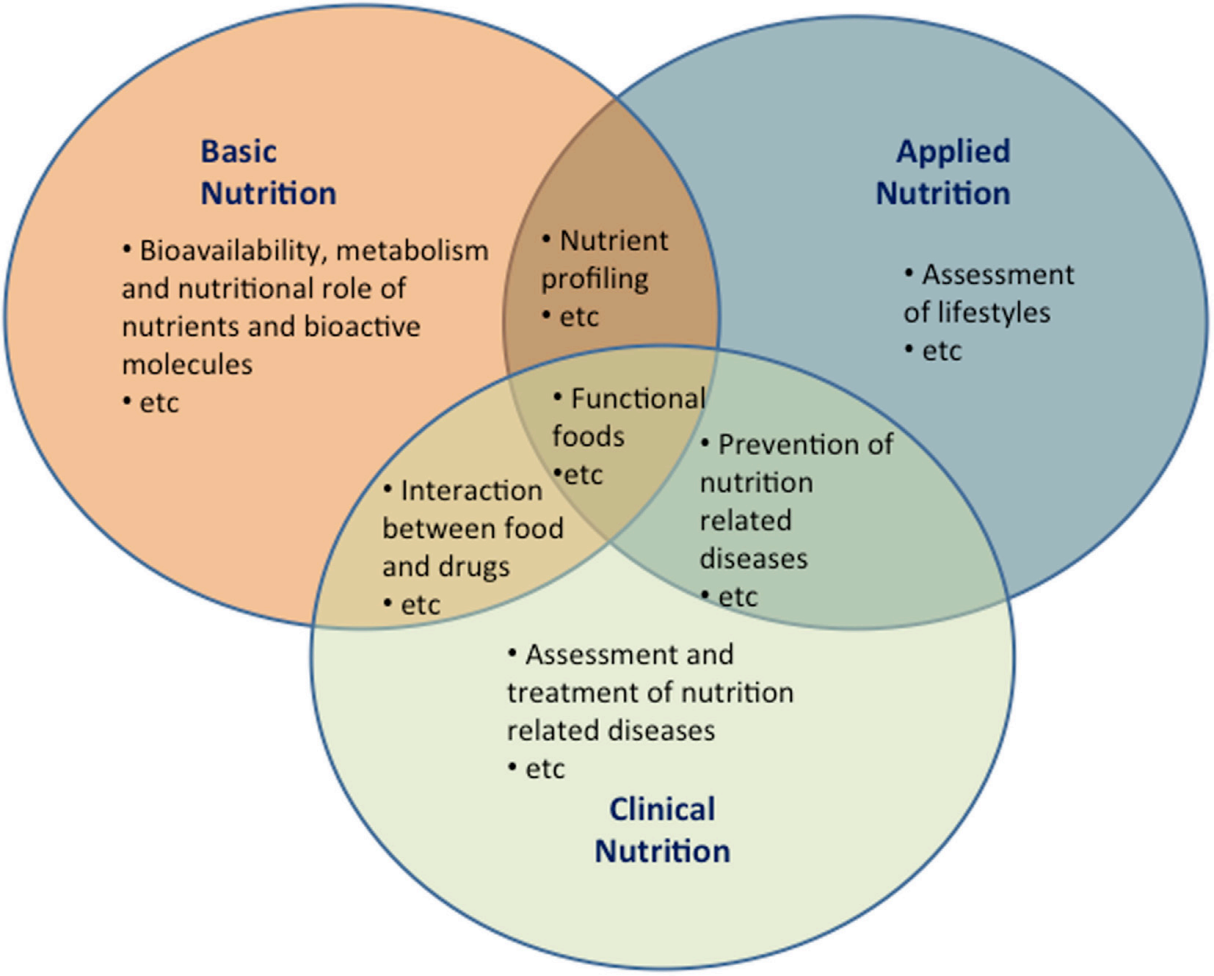
**The domains of human nutrition**. Possible areas of overlapping between the three domains of human nutrition (basic, applied, and clinical nutrition). Overlapping areas underscore the need of integration between the different professionals involved in the field of human nutrition.

Federation of the Italian Nutrition Societies has identified three main domains of human nutrition, namely, basic nutrition, applied nutrition, and clinical nutrition. These three domains have their own cultural and scientific identity, specific aims, and are clearly corresponding to professional skills. On the other hand, while being distinguished, these three domains *are and must be* closely connected and integrated both for academic training and professional activity (Figure 2).

#### Basic Nutrition

Basic nutrition is the discipline that deals with the scientific bases of human nutrition. It focuses first on the molecular, genetic, biochemical, physiological, and metabolic aspects relating to the mechanisms of action, nutritional effects, and regulation systems of food components, nutrients, and other bioactive molecules. Basic nutrition studies and characterizes the presence, bioavailability, mechanisms of action, and biochemical–physiological roles of nutrients and bioactive molecules.

#### Applied Nutrition

Applied nutrition is the discipline dealing with the relationships between nutrition and health/well-being of the general population, at all ages. Aims of applied nutrition include the promotion of healthy eating, the prevention of malnutrition (over- and undernutrition, selective deficits of nutrients) and of nutrition-related diseases, and the study of the dietary habits of special groups and communities of healthy individuals. Applied nutrition concentrates on improving the nutritional quality of foods, on primary prevention, surveillance, and nutritional epidemiology, establishing reference values and guidelines for healthy eating, dietary education, and collective catering for the healthy population.

#### Clinical Nutrition

Clinical nutrition is the medical discipline focusing on assessing, preventing, diagnosing, and treating malnutrition (over- and undernutrition, selective deficits of nutrients) related to acute and chronic diseases at all ages. Clinical nutrition deals with the metabolic changes in single patients or groups and in individuals at risk of nutritional impairment. Clinical nutrition employs validated strategies for the evaluation of nutritional status, nutritional therapy and rehabilitation, behavioral and pharmacological approaches, such as dietary intervention for specific pathologies, artificial nutrition, or selective supplementation with specific nutrients.

### Areas of Knowledge Pertinent to Human Nutrition

Federation of the Italian Nutrition Societies has examined the areas of knowledge pertinent to human nutrition. Thirty-two items were identified, which in most cases are shared with other cultural–scientific fields, such as molecular biology, biochemistry, physiology, hygiene, and food technology. These areas of knowledge were attributed to one or more of the domains (basic, applied, clinical) of human nutrition. Finally, the areas of knowledge were ranked considering their diverse importance for academic training in the different domains (basic, applied, clinical) of human nutrition. The required levels of knowledge were ranked as basic (+), intermediate (++), and high (+++) (Table 1). Finally, the study group proposed the attribution of the different areas of knowledge to the degree courses where training in human nutrition is deemed necessary (e.g., schools of medicine, biology, nursing, etc.) (see Table S1 in Supplementary Material).

**Table 1:** **Importance of the areas of knowledge in the three domains of human nutrition**.

Areas of knowledge	Domains		
	Basic	Applied	Clinical
Genetic and molecular basis of metabolism	+++		+
Bioavailability, metabolism, and nutritional role of nutrients and bioactive molecules	+++	+	+
Food composition (nutrients and bioactive molecules)	+++	+	+
Nutrient profiling	+++	+++	+
Effects of transformation and preservation on the nutritional characteristics of food	++	+++	+
Development and utilization of functional foods	++	+++	++
Food and nutrition safety (best practice, novel foods, contaminants, additives, preservatives)	+	+++	+
Food allergies and intolerances	+	+	+++
Assessment of nutritional status	++	++	++
Clinical diagnosis of nutritional status			+++
Nutritional surveillance		+++	++
Nutritional epidemiology		+++	++
Physiological nutrition at different ages		+++	+
Nutrition during pregnancy and breast-feeding		+++	+
Sports nutrition		+++	+
Assessment of dietary adequacy	+	+++	+
Assessment of lifestyles		+++	+
Assessment of eating habits, behavior, and food choices	++	++	+
Assessment of interactions between food and drugs	++	+	+++
Primary and secondary prevention of nutrition-related diseases		+++	++
Tertiary prevention of nutrition-related diseases		+	+++
Promotion of healthy eating and dietary education		+++	+
Communication and dissemination of information in nutrition		+++	+
Commercial catering and food services		+++	+
Catering and food services in health-care settings (hospital, nursing homes)		+	+++
Training for food business operators		+++	
Assessment and nutritional treatment of eating disorders			+++
Assessment and nutritional treatment of nutrition-related diseases			+++
Assessment and nutritional treatment of inborn metabolic disorders			+++
Artificial nutrition (enteral and parenteral)			+++
Pharmaconutrition and nutraceuticals			+++
New food production technologies (genetically modified foods, nanotechnologies, etc.)	+++	++	+

*“Amount” of knowledge required: basic, +, intermediate, ++, high, +++*.

## Discussion

In view of the various objective critical points and lack of clarity on this topic, FeSIN has issued the present position paper setting out a series of criteria and guiding concepts in the attempt to improve academic training in human nutrition. Considering the vastity of this broad and multidisciplinary matter, the working group concentrated on the identification and definition of human nutrition domains and on the analysis of the areas of knowledge that could be attributed to basic, applied, and clinical nutrition, respectively. The list of areas of knowledge and the rank attributed to the different areas of knowldege are the result of a discussion between experts of different backgrounds and resulting in different sensibility. This may account for some difference in judgment and opinion on individual aspects of human nutrition. This position paper also offers a first step toward a better organization academic training in postgraduate courses like Master and Residency courses (see details in Supplementary Material).

The relevant issue of the low “nutritional culture” linked in particular to an insufficient academic training for HCPs has been long described. In different studies, most of resident physicians and general practitioners consider themselves not adequately trained to provide nutrition counseling (23, 24). These gaps in knowledge were found to be positively correlated with the level of education the physicians received and self-perceived education was found to be associated with higher levels of knowledge (25). Although 98% of medical schools in the US reports nutrition as a component of medical education, in fact a nutrition curriculum is not identifiable in most schools while the effect on clinical skills in medical schools that do include nutrition has not been evaluated (26). The compelling need to improve nutritional knowledge and skills among HCP is substantiated by a number of studies. Friedman and colleagues (27) indicated that the “mission to increase the number of physician nutrition experts at the undergraduate and postgraduate levels requires an analysis of the current status of nutrition education in U.S. medical schools.” Moreover “a survey of past efforts at solving the problem of teaching the essentials of nutrition to medical students, and recommendations aimed at initiating helpful short- and long-term programs to create greater numbers of physician nutrition experts” is deemed necessary. The authors also wisely highlight the necessity to integrate courses both horizontally and vertically, connecting the basic sciences and clinical medicine. They finally proposed short- and long-term recommendations. In particular, they advise that a 1-year course emphasizing “essentials of nutrition” should be offered nationwide to committed individuals among all specialties and that nutrition topics should be progressively introduced in academic training.

In the UK, the National Nutrition Task Force developed in 1990s a core curriculum for health professionals. In this curriculum, different points considering the principles of food science, public health, and clinical nutrition were considered (28).

The University of Colorado School of Medicine in 2001 developed a comprehensive nutritional curriculum considering that nutrition content should be broad in nature, vertically integrated from preclinical to clinical and postgraduate training, while active learning (e.g., “learning by doing”) needed to be practiced whenever possible. The integration of nutrition into the curriculum needs to consider the necessity to identify a core group of committed faculty to advocate for nutrition together with a network considering other elements of the existing curriculum. By this way, nutrition content will be incorporated in clinical training without necessarily adding time (29).

In 1993–1997 at the University of Arizona, a research program was built to verify if an integrated nutrition curriculum (characterized by doubling the total hours of required instruction in the medical curriculum—35 vs 75 h) could improve the performance on nutrition-oriented clinical examinations of medical school classes. The implementation of the curriculum allowed an improvement of the Objective Structured Clinical Examination nutrition score (41.7 ± 0.9% vs 50.6 ± 1.1%) and the percentage of students who reported that the amount of nutrition taught during medical school was inadequate decreased (from 68.4 to 11.5%) (26). In 1998, the Nutrition Academic Award (NAA) recipients developed the *Nutrition Curricular Guide for Training Physicians*. The aim of this plan was to incorporate clinical guidelines into physician practice skills, to create educational and assessment practice tools, and to evaluate curricula, materials, and teaching tools. The NAA is a 5-year grant awarded to a network of 21 US medical schools in the United States. The National Heart, Lung, and Blood Institute’s program gave emphasis on the prevention of cardiovascular diseases, obesity, diabetes, and other chronic diseases to encourage the development and enhancement of medical school curricula to amplify opportunities for students, residents, fellows, faculty, and practicing physicians to learn nutrition principles and clinical practice skills. Finally, learning materials (curricular guide, training tools) were provided to improve education of HCPs in medical schools and graduate programs (30).

The Need for Nutrition Education/Innovation Programme is an independent education and evaluation program, developed at the University of Cambridge (UK), that aims to equip “tomorrow’s doctors” with clinically relevant, foundation nutrition and public health knowledge to enhance nutrition care in health-care settings. The initiative includes a vertical, spiral approach during the clinically focused years of the Cambridge undergraduate and graduate medical degrees. The success of the nutrition education initiative was attributed to three factors including the leadership and advocacy skills of the nutrition academic team, the variety of teaching modes, and the multidisciplinary approach to teaching (22).

The Boston University of Health and Rehabilitation developed a model to integrate nutrition medicine at the medical school based on three main actions: to improve medical students’ education, an approach considering case- and practice-based learning in classroom and clinical setting together with extracurricular and virtual training was used. The results of the study showed that, during teaching period, most objectives related to nutrition medicine were covered. Moreover, new opportunities for student leadership and partnership with other health professionals were provided by extracurricular activities (31).

Taken together, the available literature indicates that nutrition education can be comprehensively integrated into medical training even without additional time or financial resources. For this purpose, a vertical integration of key principles across preclinical and clinical courses was proposed into postgraduate education. Moreover, adult active teaching methods and nutrition mentors need to be implemented in the training process to confirm the relevance of nutrition in medicine (29, 32).

Based on the available literature and on the within-the-group discussion, FeSIN has elaborated a number of statements on academic training in human nutrition which could be adopted worldwide (Table 2).

**Table 2:** **Federation of the Italian Nutrition Societies position statements on academic training in human nutrition**.

human nutrition encompasses three main domains, namely, basic, applied, and clinical nutrition, each with its own cultural, scientific, and professional identity training in nutrition in health-care degree courses appears inadequate worldwide inadequate training in human nutrition of health-care professionals (HCPs) has negative impact on public health and health-care costs teaching of human nutrition should be integrated in the training goals for first-level and master’s degree courses for HCP training in human nutrition should provide the adequate knowledge, competences, and skills and for the different HCPs involved in the field of human nutrition training in human nutrition should be given adequate number of credits for basic, applied, and clinical nutrition

In conclusion, we believe that the present paper may contribute to positive outcomes within the human nutrition community. In particular, the identification and definition of the three domains of human nutrition, namely, basic, applied, and clinical nutrition, represents a significant advancement in the attempt to more clearly define the cultural identity of human nutrition in an academically- and professionally-oriented perspective. It is conceivable that, based on the implementation of knowledge-competences-skills trajectories in the different areas and domains of human nutrition, a better, pro-active, cost-effective integration of the professionals involved in this field will eventually occur.

## Author Contributions

All authors participated in the conception of the position paper.

## Conflict of Interest Statement

The authors declare that the research was conducted in the absence of any commercial or financial relationships that could be construed as a potential conflict of interest. The reviewer MC and handling editor declared their shared affiliation, and the handling editor states that the process nevertheless met the standards of a fair and objective review.
