# Supplementary material for: The Domains of Human Nutrition: The Importance of Nutrition Education in Academia and Medical Schools
Source: Front Nutr. 2017 Feb 22;4:2. doi: 10.3389/fnut.2017.00002 (PMC5320893; doi:10.3389/fnut.2017.00002)
Supplement: Supplementary file 1 [file Table_1.DOCX]

**Supplementary material**

The Italian Academic training system

The Italian Ministry for the University and Scientific and Technological Research (MIUR), with the Ministerial Decree no. 509 (3^rd^ November 1999) set a far-reaching reform of the Italian University system, with the aim of unifying it with European University training systems and qualifications. Further innovations were introduced by Ministerial Decree 270, on 22^nd^ October 2004. This reorganization took place within the framework of the Bologna Declaration, signed in Bologna in 1999 by 29 EU Ministers of Education with the aim of creating a European space for higher education and promoting student mobility (1-10).

Finally, Italian Universities award the following qualifications: Bachelor’s Degrees, Post-graduate Degrees, Research Doctorates, Specializations (Residency Training Programs) and First and Advanced level Master Degrees:

1. Bachelor’s Degree Courses (Laurea, undergraduate degree) are aimed at providing students with an appropriate mastery of general scientific methodologies and contents together with the acquisition of specific professional knowledge. These courses last three years. To get the Bachelor’s Degree students have to obtain 180 credits (60 credits per year);
2. Post-graduate Specialised Degree Courses (Laurea Magistralis) are aimed at providing students with advanced qualified training leading to a high qualified professional activity in specific fields. These Courses last at least two years after the first Degree course (120 credits);
3. Specialization Courses (Residency Training Programs) are aimed at providing post-graduate students with knowledge and abilities required by particular professional activities;
4. Research Doctorate Courses are directed at the in depth study of research methodology and advanced scientific preparation in different scientific sectors;
5. University Master Courses represent scientific specialization courses subsequent to the obtainment of a University Degree (1^st^ level Master) or a Post-graduate Specialised University Degree (2^nd^ level Master).

The University Reform considers the ECTS (European Community Course Credit Transfer System) that was developed by the Commission of the European Union in order to provide common procedures to guarantee academic recognition of studies abroad, providing a way of measuring and comparing learning achievements, and transferring them from one institution to another. ECTS credits are a value allocated to course units to describe the student workload required to complete them. In the ECTS, 60 credits represent the workload of a year of study. Each ECTS credit (equivalent to 25 hours) comprises the hours a student spends in individual study, face-to-face lessons, workshop or laboratory work, exercises and practice, etc. ECTSs are intended to allow students greater flexibility in designing their study plans, to facilitate mobility between universities – in Italy and elsewhere, and to simplify recognition of Italian training and university qualifications in other countries.

For each degree class, training is grouped under disciplinary headings, each made up of different Scientific and Disciplinary Sectors which generate specific teaching plans. Under this classification, there are a total of 370 scientific disciplines grouped into 14 distinct and clearly identifiable areas. Each university is free to select its own study courses, for each Degree/Master’s Degree class. These courses must all be aimed at the goals for students in that class and the corresponding training needs, and the qualifications earned all have the same legal standing.

Degrees/Master’s Degrees usually have a range of training goals, implying the need for a series of skills – knowledge, ability, competences – in relation to the potential career outlets. Each university starts up its study courses on the basis of nationally approved specifications, and on its internal curriculum. Each university is responsible not only for selecting study courses, but also for ensuring they meet the specific needs for certain further training areas. As a consequence, the study courses for the same Degree/Master’s Degree classes may differ substantially in the subject matters covered and how the teaching is organized.

### Academic training in human nutrition

Training in human nutrition is a complex process, mainly because of its multidisciplinary nature, which in turn reflects into the differences between the indications for the various degrees. Teaching in human nutrition is an essential part of some study courses, like the degree courses in Dietetics or in Human Nutrition Sciences; it may be specified as one of the training objectives in courses such as the degree course in Food Science and Technologies, or the postgraduate course in Pharmacy and Industrial Pharmacy. In other cases, the nutritional aspects may be set out less clearly, but can still be identified on the basis of direct and indirect considerations. This is the case, for instance, of the postgraduate course in Biology. In other degree courses, training in human nutrition may be a reasonable, logical requirement, without being specifically indicated in the Ministerial Decree currently in force. Examples include the degree courses for health and dental professions, motor sciences, biotechnologies and agrarian science.

On the whole, however, as stated before, FeSIN considers that training in human nutrition is insufficient in many study courses today, with inadequate goals, encompassing poorly divided areas of knowledge-ability-competences. FeSIN therefore considers it important that training in human nutrition should be included not only among basic study topics, but also among those aimed at specific careers. This can be achieved by specific courses, or as modules forming part of integrated courses, with explicitly assigned university ECTS credits.

In view of the multidisciplinary nature of the topic, FeSIN has summarized the subject matters that must be covered in the various study courses forming the core of training in human nutrition, indicating the minimum number of specific ECTS credits to be earned during the course. The second aim of the present paper was the formulation – however summary – of the indispensable, fundamental cultural ‘core’ on human nutrition knowledge that must be taken into account in the curricula and regulations of the different study courses. An open issue for each course is the definition of the subject matter required in terms of knowledge-ability-competences, and this refers to a close cooperation between universities, scientific societies, Ministry of health, professional orders and all other parties possibly involved. ECTS credits for degree courses in Dietetics or in Human Nutrition Sciences are not specified as these are all by definition nutrition-oriented (Supplementary Table 1).

**References**

1. The European Qualifications Framework (EQF).

http://ec.europa.eu/education/lifelong-learning-policy/doc44_en.htm

1. Decreto Ministeriale 3 novembre 1999. Regolamento recante norme concernenti l’autonomia didattica degli atenei. GU n.2 del 4 gennaio 2000
2. **Decreto Ministeriale 22 ottobre 2004. Modifiche al regolamento recante norme concernenti l'autonomia didattica degli atenei, approvato con decreto del Ministro dell'università e della ricerca scientifica e tecnologica 3 novembre 1999, n. 509.** GU n. 266 del 12 novembre 2004
3. Decreto Ministeriale 16 marzo 2007Determinazione delle classi delle lauree. GU n. 153 del 6 luglio 2007
4. Decreto Ministeriale 16 marzo 2007. Determinazione delle classi delle lauree magistrali. GU n. 155 del 9 luglio 2007
5. Decreto Interministeriale 19 febbraio 2009. Determinazione delle classi delle lauree delle professioni sanitarie. GU n. 119 del 25 maggio 2009
6. Decreto Ministeriale 8 gennaio 2009. Determinazione delle classi delle lauree magistrali delle professioni sanitarie. GU n. 122 del 28 maggio 2009
7. Decreto 29 luglio 2011. Determinazione dei settori concorsuali, raggruppati in macrosettori concorsuali, di cui all'articolo 15, legge 30 dicembre 2010, n. 240. G.U. n. 203 del 1 Settembre 2011
8. Ministero dell’Istruzione, dell’Università e della Ricerca. Dipartimento per l’università, l’alta formazione artistica, musicale e coreutica e per la ricerca. Quadro dei Titoli Italiani. Rome; CIMEA, 2010. www.quadrodeititoli.it
9. The European Higher Education Area. <http://www.ehea.info>

**Supplementary Table 1: Areas of knowledge related to human nutrition to be considered for inclusion in the different study courses**

|  | **Degree Courses** | | | | | |
| --- | --- | --- | --- | --- | --- | --- |
|  | **Medicine^2^** | **Dietetics^1^** | **Human nutrition^1^** | **Biology (applied to food science and nutrition)^2^** | **Dentistry and dental prosthetics^2^** | **Pharmacy and industrial pharmacy^2^** |
| Credits | 14 |  |  |  | 3 | 12 |
| Biochemistry of nutrition | + | + | + | + |  | + |
| Genetic and molecular basis of nutrition |  |  | + |  |  |  |
| Nutrients and bioactive molecules | + | + | + | + | + | + |
| Nutritional evaluation of foods and diet |  |  | + | + |  |  |
| Physiological nutrition at different ages | + | + | + | + | + | + |
| Nutrition during pregnancy and breast-feeding | + | + | + | + | + | + |
| Promotion of healthy lifestyles | + |  |  | + |  | + |
| Dietary education |  |  |  |  | + |  |
| Dietary education and  nutritional surveillance |  | + | + | + |  |  |
| Communication and dissemination of information in nutrition |  |  | + |  |  |  |
| Screening and assessment of nutritional status in physiological conditions |  |  | + | + |  |  |
| Screening and assessment of nutritional status in physiological and pathological conditions |  | + |  |  |  |  |
| Assessment, diagnosis and  follow-up of nutritional  status in physiological and  pathological conditions | + |  |  |  |  |  |
| Nutritional quality of  foods and dietary habits |  | + |  |  |  |  |
| Assessment of eating  habits and choices in  physiological  conditions |  |  | + | + |  |  |
| Assessment of eating  habits and choices in  physiological and  pathological conditions |  | + |  |  |  |  |
| Primary prevention of  nutrition-related diseases |  |  | + | + |  |  |
| Primary, secondary and  tertiary prevention of  nutrition-related diseases | + | + |  |  |  |  |
| Nutritional therapy for  metabolic and nutritional  disorders | + |  |  |  |  |  |
| Dietetic aspects of  metabolic and nutritional  pathologies including food  allergies and intolerance |  |  | + |  |  |  |
| Dietetic aspects of  metabolic and nutritional  pathologies including  eating behavior disorders,  food allergies and  intolerance |  | + |  |  |  |  |
| Nutritional therapy for  chronic-degenerative and  oncologic pathologies, and  highly prevalent  conditions including  eating behavior disorders,  food allergies and  intolerance | + |  |  |  |  |  |
| Principles of artificial  nutrition (enteral and  parenteral) |  | + |  |  |  |  |
| Principles of artificial  nutrition (enteral and  parenteral), nutritional  pharmacologiy and  nutraceuticals |  |  |  |  |  | + |
| Management of artificial  nutrition (enteral and  parenteral), nutritional  pharmacologiy and  nutraceuticals | + |  |  |  |  |  |
| Oral nutritional  supplements and foods for  special purposes |  |  |  |  |  | + |
| Collective catering in  healthcare settings, and  nutritional security | + |  |  |  |  |  |
| Collective catering:  institutional, commercial  and in healthcare  settings |  | + | + | + |  |  |
| Food safety |  | + | + | + |  |  |
| Effects of technological transformation and preservation on the nutritional characteristics of food |  | + | + | + |  |  |
| Development and use of functional foods |  |  | + |  |  |  |
| Interaction between drugs and nutrients/foods |  |  |  |  |  | + |

Legend: ^1^ Bachelor’s Degree Courses - Laurea; ^2^ Post-graduate Specialised Degree Courses – Laurea Magistralis

NB: ECTS credits for degree courses in Dietetics or in Human Nutrition Sciences are not specified as these are all by definition nutrition-oriented

|  | **Degree Courses** | | | | |
| --- | --- | --- | --- | --- | --- |
|  | **Pharmaceutical sciences and technologies^1^** | **Food chemistry and technologies^2^** | **Biotechnologies^1^** | **Nursing^1^** | **Nursing & obstetrics^2^** |
| Credits | **12** | **9*** | **4** | **6** | **2** |
| Biochemistry of nutrition | **+** | **+** | **+** |  |  |
| Genetic and molecular basis of nutrition |  |  |  |  |  |
| Nutrients and bioactive molecules | **+** | **+** | **+** | **+** |  |
| Nutritional evaluation of foods and diet |  | **+** |  |  |  |
| Effects of technological transformation and preservation on the nutritional characteristics of food |  | **+** |  |  |  |
| Development and use of functional foods |  | **+** |  |  |  |
| Physiological nutrition at different ages | **+** |  | **+** | **+** |  |
| Nutrition during pregnancy and breast-feeding | **+** |  | **+** | **+** |  |
| Promotion of healthy lifestysles | **+** | **+** | **+** | **+** |  |
| Promotion of healthy lifestysles in particular in athletes |  |  |  |  |  |
| Primary prevention of nutrition-related diseases |  | **+** |  |  |  |
| Principles of assessment of nutritional status in physiological conditions |  |  |  |  |  |
| Screening of nutritional status and food intake in physiological and pathological conditions |  | **+** |  | **+** |  |
| Nutritional therapy for chronic-degenerative and  oncologic pathologies, and highly prevalent conditions  including eating behavior disorders, food allergies and  intolerance |  |  |  | **+** |  |
| Principles of artificial nutrition (enteral and parenteral),  nutritional pharmacologiy and nutraceuticals | **+** |  |  |  |  |
| Principles of artificial nutrition (enteral and parenteral),  and management of infusion protocols |  |  |  | **+** |  |
| Organization and management of enteral and parenteral  artificial nutrition and management of infusion  protocols (hospital and homecare) |  |  |  |  | **+** |
| Oral nutritional supplements and foods for special purposes | **+** |  |  |  |  |
| Collective catering: institutional and commercial |  | **+** |  |  |  |
| Collective catering in healthcare settings (principles)  and nutritional security |  |  |  | **+** |  |
| Organization and management of collective catering in  healthcare settings and nutrition security |  |  |  |  | **+** |
| Role of nutrition in the prevention and treatment of  pressure ulcers (bedsores) |  |  |  | **+** |  |
| Interactions between drugs and nutrients/foods | **+** |  |  |  |  |

Legend: ^1^ Bachelor’s Degree Courses - Laurea; ^2^ Post-graduate Specialised Degree Courses – Laurea Magistralis

* 20 credits in the specific curricula for collective catering

|  | **Degree Courses** | | | | | |
| --- | --- | --- | --- | --- | --- | --- |
|  | **Motor sciences^1^** | **Sciences and techniques of sport^2^** | **Obstectrics^1^** | **Phsyiotherapy^1^** | **Dental hygiene^1^** | **Health practice and prevention^2^** |
| Credits | **4** | **4** | **4** | **4** | **2** | **2** |
| Nutrients and bioactive molecules | **+** | **+** | **+** | **+** | **+** | **+** |
| Physiological nutrition at different ages | **+** |  |  | **+** | **+** | **+** |
| Nutrition during pregnancy and breast-feeding |  |  | **+** |  |  |  |
| Promotion of healthy lifestysles |  |  | **+** | **+** | **+** | **+** |
| Promotion of healthy lifestysles in particular in athletes | **+** | **+** |  |  |  |  |
| Primary prevention of nutrition-related diseases |  |  |  |  |  |  |
| Principles of assessment of nutritional status in physiological conditions | **+** | **+** |  |  |  |  |
| Screening of nutritional status and food intake in physiological and pathological conditions |  |  | **+** | **+** |  |  |
| Principles of nutritional therapy for malnutrition |  |  |  | **+** |  |  |

Legend: ^1^ Bachelor’s Degree Courses; ^2^ Post-graduate Specialised Degree Courses
